# Effects of Eucalyptus Plantations on Detritus, Decomposers, and Detritivores in Streams

**DOI:** 10.1100/tsw.2002.193

**Published:** 2002-04-30

**Authors:** Manuel A.S. Graça, Jesus Pozo, Cristina Canhoto, Arturo Elosegi

**Affiliations:** Departamento de Zoologia, Universidade de Coimbra, Largo Marquês de Pombal, 3004-517 Coimbra, Portugal; 2Departamento de Biología Vegetal y Ecología, Universidad del País Vasco, Apdo. 644, 48080 Bilbao, Spain

**Keywords:** decomposition, afforestation

## Abstract

Vast areas of the Iberian Peninsula are covered by monocultures of the exotic tree *Eucalyptus globulus*. Given that (1) leaf litter produced in the riparian areas is the main energy source for small streams, and (2) trees differ in their nutrient content, chemical defenses, and physical attributes, eucalypt plantations have the potential to affect the biology of streams. Research teams from the University of Coimbra and the University of the Basque Country have been addressing the potential effects of eucalypt plantations at several levels of study. Here we review the main conclusions of these investigations.

Eucalypt plantations produced less litter than some deciduous forests. However, there were marked differences in timing of litterfall: litter production peaked during autumn in deciduous forests, whereas in the eucalypt forests it tended to peak in summer and to be more evenly distributed throughout the year. Despite these differences, the average standing stock of organic matter was higher in the eucalypt than in the deciduous forest. This may be attributed to (1) the occurrence of spates or heavy rain in autumn, the period of maximum litter fall in deciduous forests, and (2) bark accumulation in eucalypt forests. Because of differences in leaf composition, the nutrient input in eucalypt forests seems to be lower than in deciduous forests. The rate of decomposition of eucalypt leaves was strongly dependent on nutrients in the water: in nutrient-poor waters it was slower than that of most other leaf species, whereas in nutrient-rich waters it can be as fast as alder – a fast-decaying species.

The biomass and cumulative diversity of aquatic hyphomycetes colonizing leaves did not differ between eucalypt and other native leaf species, but fungal sporulation generally peaked 2 weeks later on eucalypt leaves. This lag disappeared when lipids (but not polyphenolics) were chemically removed from eucalypt leaves. Similarly, addition of eucalypt oils to culture media retarded or suppressed fungal growth. Streams bordered by *Eucalyptus* had lower diversity of fungal spores (but similar spore densities) in Portugal; less consistent patterns were found in similar experiments in Spain.

*Eucalyptus* leaves proved to be poor food for shredders. Under laboratory conditions leaves of *Eucalyptus* ranked low in food selection experiments using native shredders. The same shredders failed to grow and died when fed exclusively eucalypt leaves. The removal of oils from eucalypt leaves resulted in increased feeding rates, whereas the transfer of oils to alder leaves resulted in decreased feeding rates.

The effect of eucalypt plantations on stream invertebrate communities is not very consistent. In nutrient-poor waters, fewer invertebrates colonized eucalypt than alder leaves, but this effect was mitigated after a microbial conditioning period in nutrient-rich waters. Portuguese streams bordered by *Eucalyptus* had lower numbers of invertebrates than streams surrounded by deciduous forests. In Spanish streams differences were less marked and nonexistent when looking at the composition of the communities, which change more from year to year than from site to site. Most of the eucalypt streams studied in Portugal and Spain dried up in summer, a fact that might reflect an increase in soil hydrophobity produced by *Eucalyptus* plantations.

The very short planting-to-harvest period of eucalypt plantations results in additional impacts, such as soil loss, siltation of streams, or reduced amounts of woody debris in stream channels, which affects their capacity to retain leaf-litter, as well as the availability of habitat for invertebrates and fish. The studies by the Portuguese and Spanish research teams confirm the importance of maintaining riparian buffer strips to reduce human impact on streams and rivers.

## RIPARIAN VEGETATION PROVIDES LOW ORDER STREAMS WITH ENERGY IN THE FORM OF ORGANIC MATTER

Forests produce large amounts of litter in the form of leaves, fruits, flowers, and wood. The amount of litterfall varies with forest type, climate, season, and year. In a recent review, Abelho[1] computed an average of 602 g (dry mass) of forest litter production/m$^2$/year (maximum of 2789 g; 38 locations worldwide). For comparison, several European streams averaged 530 g/m$^2$/year (maximum 1719 g)[2]. Some of this organic matter reaches streams and rivers. Therefore, riparian trees provide streams with abundant organic matter and, simultaneously, limit light penetration and consequently primary production.

The unidirectional flow of streams tends to wash away leaves and other particles of organic matter, but since low-order streams are typically narrow and shallow, leaves are efficiently retained in pools, riffles, and by structures projecting from the stream bottom such as wood and twigs. Although retention depends on the channel structure and stream hydraulics, several studies have shown that 90% of the leaves falling in low order streams (< $4^h$) may be trapped within 10–80 m[3,4,5]. The standing stock of organic matter can be as high as 1914 g ash-free dry mass (AFDM) per square metre in low order streams[1]. Allochthonous organic matter is therefore considered to be the main energy source for streams running through forests[1,6].

### Leaves Entering the Streams are Incorporated Into Secondary Production

Leaves in streams are decomposed by the joint actions of physical factors (leaching of soluble compounds and physical fragmentation) and biological breakdown (mediated by decomposers and detritivores). Although fungi and bacteria colonize leaves before they reach rivers, aquatic hyphomycetes rapidly replace the terrestrial decomposers (e.g., [7]). The biomass of aquatic hyphomycetes may account for up to an estimated 8–16 % of the total leaf mass[8,9,10,11,12]. Aquatic hyphomycetes produce enzymes capable of breaking down plant structural polysaccharides[13,14,15], whose subunits are then incorporated into fungal biomass[16].

Allochthonous organic matter, either in the form of coarse particulate organic matter (CPOM) or fine particulate organic matter (FPOM), is also a food resource for invertebrates, especially after fungal colonization [17,18,19]. Invertebrates feeding on CPOM are known as shredders, and they have a key role promoting the production of FPOM, which can be consumed by collectors and filterer invertebrates. Litter is therefore incorporated into detritivores biomass and transferred to higher trophic levels[16,19,20,21,22,23,24].

### Changes in Forest Composition Can Affect Aquatic Biota

The timing of leaf abscission, nutrients, and chemical defenses of leaves differ among tree species. Chemical compounds presumably involved in plant defenses against bacterial and fungal infection may remain active after senescence, interfering with microbial enzymes and invertebrate feeding, and thereby slowing down decomposition. Leaves rich in nutrients such as nitrogen tend to decompose quickly and are preferred by shredders, whereas leaves containing large amounts of chemical defenses decompose slowly and are less palatable to shredders[25,26].

Given the unequivocal evidence that low-order streams derive most of their energy from leaves originating from the riparian zone and that leaves of different species vary in their quality, changes in the riparian forest have the potential to strongly affect stream biota. For example, Molles[27] reported that streams under post-fire aspen forests in New Mexico had less organic matter and fewer shredders than streams under unburnt pine forest. Murphy and Giller[28] reported a higher density of shredders and benthic detritus in streams running through conifer plantations in southwest Ireland than in streams running through deciduous forests. Stout et al.[29] found that streams running through a mature hardwood forest in Southern Appalachians had higher standing crop of detritus, lower proportion of fast-decomposing leaves and lower shredder production than streams running through young/disturbed forests. These and other studies highlight the multiple mechanisms by which changes in riparian vegetation can affect stream biota.

## CONCERN ABOUT THE IMPACT OF LARGE *EUCALYPTUS* PLANTATIONS

*Eucalyptus globulus* Labill. is an Australian tree introduced to the Iberian Peninsula around 1829. Today, large areas of Portugal and Spain are covered with monocultures of this species. In central Portugal, eucalypt plantations occupy over 21% of the area, replacing native pine and deciduous forest[30], and in many areas of northern Spain the cover of eucalypt plantations is even greater[31]. Since allochthonous organic matter is so important for stream energetics, eucalypt plantations are likely to affect stream ecology.

Graça[32] suggested that eucalypt afforestations might affect aquatic communities because of seasonal differences in litterfall reported from Australian eucalypt forests, and because eucalypt leaves have been referred to as being of poor quality by Australian researchers[33]. It is also plausible that decreased litter diversity in plantations, and the antibiotic properties of *Eucalyptus* oils could interfere with microbial decomposition and invertebrate feeding. Research teams from the University of Coimbra and the University of the Basque Country have been addressing the potential effects of eucalypt plantations at several levels of study.

## Eucalypt Plantations Change the Timing of Litter Production…

The first objective was to compare litter production in *E. globulus* plantations and in deciduous forests. In central Portugal, mixed deciduous forests (with abundant *Castanea sativa*, *Prunus* spp. and *Quercus* spp.) and eucalypt plantations did not differ in the yearly amount of organic matter production[34]. That was not the case in Spanish studies; there, eucalypt plantations had 32% lower production of leaf litter than deciduous sites dominated by oak (*Quercus* spp.)[35]. Nevertheless, recent research (Molinero and Pozo, unpublished) suggests that caution is necessary when extrapolating results of litter production from a few plots, since differences between stands as well as interannual fluctuations can be important.

The seasonality of litterfall was significantly altered in eucalypt plantations at both Portuguese and Spanish study sites (Fig. 1). In deciduous forests, litter production occurred mainly in late autumn (October to December). Conversely, in eucalypt forests litterfall was evenly distributed throughout the year and/or peaked in summer (July–August)[34,35,36]. This pattern of high summer litter production was also described for Australian eucalypt forests[37].

The potential impact of changed litterfall patterns may be amplified by the hydrological and temperature regimes. At deciduous sites litterfall occurs when temperatures decrease and the precipitation tends to be high. In contrast, litterfall in eucalypt forests coincides with the year's highest temperatures and low precipitation[38].

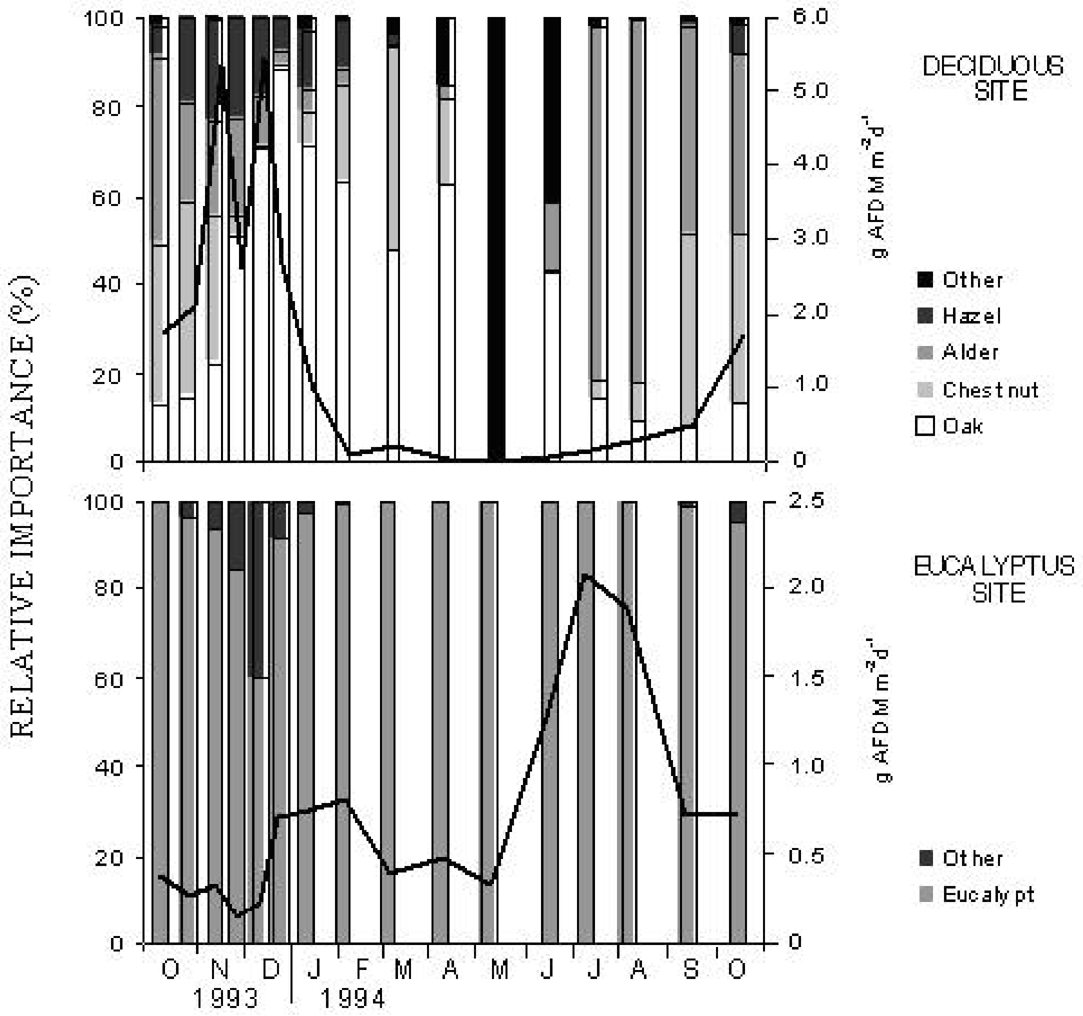

**FIGURE 1.** Temporal variation in leaf-litter (line) and in the relative importance of the main leaf species (bars) entering the streams (one deciduous and one eucalypt plantation) in the Agüera basin (northern Spain).

### … and Benthic Storage is Highly Affected

Streams running through eucalypt forests had higher amounts of benthic particulate organic matter than streams running through deciduous forests. For instance, in the Portuguese study, a mean of 309 g $m^{-2}$ in *Eucalyptus* streams contrasted with 67 g $m^{-2}$ (AFDM) in deciduous forest streams[34]. The difference may be a consequence of (1) higher retention of eucalypt forests streams caused by the accumulation of large amounts of highly refractory bark strips of eucalypt, and (2) the coincidence of peak litter production with low water level in streams. Whatever the mechanism, leaf-litter budgets differ between the two stream types, resulting in lower processing efficiencies in deciduous streams because more leaf-litter is transported downstream[39].

Eucalypt leaves have low nitrogen levels compared to many deciduous leaves[40,41] and thus contribute less nutrients to the streams[42]. According to Pozo et al.[35], differences in leaf chemistry resulted in a 67% decrease in nitrogen and 65% decrease in phosphorus inputs into eucalypt forest streams when compared with oak forest streams. However, mean storage of benthic particulate organic nitrogen and phosphorus did not differ between the two stream types, since *Eucalyptus* sites retained more benthic particulate organic matter[43].

## DECOMPOSITION IS DEPENDENT ON LEAF NUTRIENT CONTENT

Decomposition of *E. globulus* leaves has been investigated in Australia[44], California[40], Spain[11,41,45,46], Portugal[47,48], and India[49]. In these studies, leaves lost 50% of their mass after between 40–250 days (northern Spain), 76 days (northern Portugal), 84 days (central Portugal), 84–90 days (India), and over 90 days (California). Leaves of *E. globulus* can be therefore classified as medium to slow decomposing category[50]. Decomposition is influenced by nutrient content of leaves[51], and as Pozo et al.[11,41,45] showed, it is also strongly stimulated by increased nutrient levels in the water column (Fig. 2).

### Fungal Colonization of *Eucalyptus globulus* Leaves is Slow

There were some early speculations that aquatic hyphomycetes did not colonize eucalypt leaves. More recent studies proved this to be wrong; leaves of several species of *Eucalyptus* are colonized by a variety of aquatic hyphomycete species both in Australia[52] and in the Iberian Peninsula[47,48,53].

A common finding in Portuguese and Spanish streams was that fungal spore production from eucalypt leaves exposed in streams lagged two weeks behind that of other leaf types[47,48,53]. Cumulative spore production in Portuguese streams was higher from eucalypt than from alder leaves, and at later stages of decay, the diversity of aquatic hyphomycetes on eucalypt leaves was similar to other leaf types[47].

Possible reasons for the delayed sporulation are the presence of essential oils, polyphenolics, and the surface waxes on leaves. The role of phenols and terpenes as defensive compounds against fungi is generally accepted[54,55]. Eucalypt leaves contain large amounts of oils (up to 5% of leaf mass[56]) located in glands dispersed throughout the leaves. More than 70 compounds have been identified in the oil fraction, but nearly 80% correspond to 1,8-cineole and a-pinene[57,58]. Essential oils have antimicrobial[59,60] and antiherbivory properties, and toxic or deterrent effects have also been demonstrated [61,62].

Polyphenolics are considered to be one of the most widely distributed quantitative chemical defenses of leaves against herbivory[55] and possibly against fungi: they bind to proteins, including digestive enzymes, and therefore can block digestion. Bärlocher et al.[47] chemically extracted polyphenolics and lipids from eucalypt leaves to investigate their relative importance in the delay of fungal colonization. The extraction of polyphenolics did not stimulate sporulation; however, the

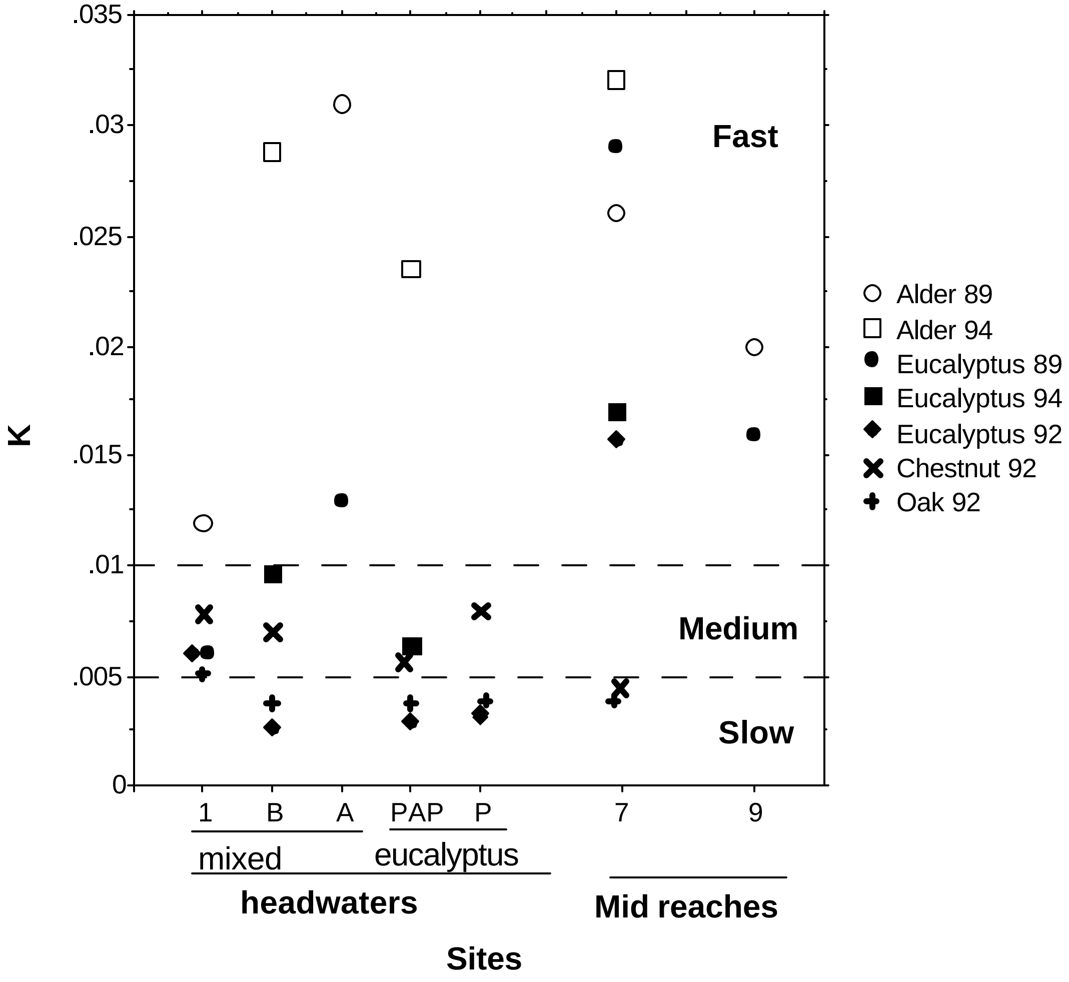

**FIGURE 2.** Breakdown rates of different leaf species incubated in headwaters and mid reaches of the Agüera stream (Spain). Note that while native deciduous leaves decay at reasonably constant, species-specific rates, irrespective of site and year, *Eucalyptus* are the slowest species in nutrient-poor headwater reaches, and among the fastest in nutrient-rich mid reaches.

chemical removal of lipids (including essential oils and waxes) resulted in a sporulation pattern similar to that on alder leaves.

Canhoto and Graça[63] suggested that fungal infection of *E. globulus* leaves is inhibited initially by a physical barrier and then by the chemical defenses. An electron microscope study on decomposing eucalypt leaves showed a waxy cuticle (physical barrier) covering the epidermis with thinner extensions lining the substomatal cavities. The cuticle of *E. globulus* was virtually intact after 5 weeks of immersion in stream, still isolating the leaf parenchyma and mesophyll from the exterior. Mycelia penetration occurred soon after immersion, but it proceeded through stoma and fissures in the leaf surface. Therefore decomposition proceeded from the mesophyll. This finding was consistent with the frequent observation that leaves of eucalypt may look intact even at an advanced stage of decomposition.

Oils and, to a lesser extent, polyphenolics may act as a chemical barrier to fungal decomposition. Frequently, the oil vesicles of the eucalypt leaves remain intact at an advanced decomposition stage. *Eucalyptus* essential oils or tannic acid added to solid culture media depressed or completely inhibited growth of selected aquatic hyphomycetes[63]. However, higher concentrations of tannic acid than oils were needed to get a significant inhibition. In those experiments one of the most tolerant species to tannic acid was *Heliscus lugdunensis,* one of the first species reported to colonize *E. globulus*[48]. The mechanism by which oils affect aquatic hyphomycetes is unclear. However, Canhoto et al.[64] showed that, *in vitro*, oils interfere with selected fungal enzymes. Consistently, *H. lugdunensis* was also relatively unaffected by high concentrations of oils.

## ***Eucalyptus*** **Plantations Affect the Assemblages of Aquatic Hyphomycetes**

Eucalypt leaves being poor substrates for aquatic hyphomycetes, how do eucalypt plantations affect fungal assemblages in the field? In the Agüera basin, (northern Spain), Chauvet et al.[53] found that the cumulative numbers of fungal species participating in the decomposition of eucalypt and alder leaves were practically the same in a stream bordered by eucalypt and in a stream bordered by a mixed deciduous forests. However, the number of fungal species present on *Eucalyptus* leaves at any given time during the processing tended to be lower at the site under *Eucalyptus*. Bärlocher and Graça[65] found that rivers running through eucalypt plantations and mixed deciduous forests did not differ in the total number of transported conidia of aquatic hyphomycetes. However, eucalyptus streams had a significantly lower number of fungal taxa than deciduous forest streams. Eucalypt streams had a distinct fungal assemblage (dominated by *Flagellospora curvula* Ingold, *Lunulospora curvula* Ingold, *Anguillospora filiformis* Greathead, and *Triscelophorus acuminatus* Nawawi). By comparison, in streams with deciduous forest *Clavariopsis aquatica* de Wild., *Tetrachaetum elegans* Ingold and *Articulospora tetracladia* Ingold dominated

Since aquatic hyphomycetes play a key role in the decomposition of leaves, it was conceivable that decreased fungal diversity could affect decomposition rates. That was not the case: eucalypt and chestnut leaves decomposed at similar rate in the species-poor eucalypt streams and in species-rich deciduous streams[65]. These findings contrast with previous results by Abelho and Graça[34], which suggested slower decomposition of both chestnut and eucalypt leaves in eucalypt rivers. However, the two studies used different experimental designs, (in the earlier experiment decomposition was expressed as the difference between initial and final mass, in the latter decomposition was measured as k).

## ***Eucalyptus*** **Leaves are a Poor Food Resource for Stream Detritivores**

If plant defenses remain active for some time after senescence, it is plausible that stream invertebrates feeding on leaves may cope with small amounts of those compounds. That seems to be the case with tipulids, slow-moving shredders of low order streams. The alkaline gut of tipulid shredders allows efficient protein digestion in the presence of tannins since phenol-protein complexes are less stable at high pH[66,67,68,69].

In the laboratory, the shredders *Sericostoma vitattum* Spencer (Trichoptera: Sericostomatidae) and *Tipula lateralis* Meig. (Diptera: Tipulidae) rejected conditioned eucalypt leaves when other leaf species, representing a range from slow to fast decomposition rates, were present. None of the shredders was able to grow on a diet of eucalypt leaves and all died before pupation[70,71].

To investigate which leaf compounds were responsible for this deterrent effect, Canhoto and Graça[63] transferred oils and polyphenoloics from eucalypt to alder leaves, which were then fed to invertebrates. The removal of oils from eucalypt leaves increased consumption (10-fold) by *Tipula lateralis,* whereas the addition of oils to alder leaves resulted in a 50% decrease of consumption. Similarly, the addition of eucalypt-extracted polyphenolics to alder leaves lowered feeding rates by *T. lateralis*. Those experiments strongly suggest a deterrent effect of eucalypt oils and polyphenolics on shredders.

In the field, eucalypt leaves will eventually disappear almost completely due to the combined effect of microbial action and invertebrate feeding. Whether or not invertebrates are able to feed on *E. globulus* leaves may depend on their size. In contrast to *T. lateralis* and *S. vittatum,* which are relatively large invertebrates, much smaller chironomidae larvae caused considerable mass loss of eucalypt leaves in the laboratory. Because of their size, the chironomids were able to selectively consume the mesophyll while leaving the oil glands intact[63].

Early research in Spain[72] reported that leaves of eucalypt were colonized by fewer invertebrates than alder leaves at sites with low dissolved nutrients. However, the difference disappeared in stream reaches with higher nutrient content. Further, extended studies on leaf breakdown showed that at the end of the incubation period, eucalypt leaves reached total invertebrate and shredder densities and biomass[11,46] comparable to those previously registered with deciduous leaves.

### Do Eucalypt Plantations Affect Invertebrate Assemblages in Streams?

Shredders are expected to be abundant in low order streams[73]. In selected central Portugal forested streams, their percentage was indeed high, comprising 41–45% of the total invertebrate biomass[74]. In contrast, shredders are less abundant in the Agüera stream (northern Spain), usually accounting for 4–30% of total invertebrate numbers, although they reached 50% of biomass in headwater reaches[75,76,77].

It is plausible that eucalypt plantations affect the assemblages of aquatic macroinvertebrates for four reasons:

1. Timing of litter inputs is altered under eucalypt plantations. Species whose larval stages occur in autumn (adapted to the timing of deciduous forests litterfall) may be at higher risk of being eliminated by lack of food.
2. Colonization of leaves by aquatic hyphomycetes is slow, and fungi are important mediators in the energy transfer from leaves to shredders.
3. *Eucalyptus* leaves are poor-quality food for invertebrates because of oil and polyphenolic content.
4. Hydrocarbonates exuded by *Eucalyptus* are known to increase soil hydrophobity[78,79], and thus, to result in more variable stream discharge regimes, with more frequent or extreme spates during storms, and to be more prone to dry out in summer.

In central Portugal Abelho and Graça[34] reported that during winter, streams running through eucalypt plantations had lower number and diversity of invertebrates than streams running through deciduous forests. Mixed streams (eucalypt forests with a deciduous riparian corridor) had an intermediate status. No comparisons were possible in summer because all but one studied eucalypt streams dried out. However, this dataset is small, and thus conclusions are far from straightforward. Indeed, comparative data from the Spanish studies do not confirm the results in Portuguese streams. In Spain *Eucalyptus* streams have apparently no lower densities of invertebrates. The difference between Portuguese and Spanish studies may be related to differences in the hydrological regimen. Whereas in the Spanish sites precipitation is higher and more evenly distributed along the year, in central Portugal rains are very seasonal.

One of the suggestions of both Spanish and Portuguese studies was that the effects of eucalypt plantations could be drastically diminished if buffer strips of native riparian vegetation are kept along rivers[34,35]. The study by Abelho and Graça[34]showed that rivers running through eucalypt forests, but with deciduous trees in the riparian zone, were more similar to deciduous forest streams than to eucalypt forest streams in terms of diversity, biomass of invertebrates and community structure.

## OTHER EFFECTS OF *EUCALYPTUS* PLANTATIONS ON THE ECOLOGY OF STREAMS

The results so far highlighted show that afforestation with *Eucalyptus* affects the amount, timing, and quality of litter inputs, which is reflected in some structural and functional properties of streams. Nevertheless, to evaluate the effects of tree plantations on fluvial systems, it is necessary to have a more comprehensive view of the spatial and temporal changes in soil uses, and especially to consider the whole harvest cycle.

*E. globulus* is a very fast-growing species, whose soft and fibrous wood is mostly appreciated for paper-pulp. This has important implications: since the preferred size for pulpwood is much smaller than the preferred size for timber, and this species grows so fast, harvest cycles are extremely short (ca. 12 years in both northern Spain and Portugal). This results in eucalypt plantations being harvested almost exclusively by clearcutting, while native deciduous species (oak, beech, or chestnut) have a much longer harvest cycle (up to 200 years for oak), and are more likely to be harvested by selective logging. Therefore, large-scale clearcuts occur in eucalypt plantations much more often than in other forests.

Large-scale clearcuts have dramatic effects on stream ecosystems. Litter inputs are expected to decrease to very low values, and the detritus-based community is likely to be replaced by one based on autochthonous (algal) primary production[80]. Thus, large clearcuts will drastically change the invertebrate community[81,82,83] and fish communities[84,85]. This situation, reported for many different kinds of streams, will revert to the original status as the next generation of trees closes its canopy. In the case of eucalypt trees, the cycle of algal- to detritus-based communities is likely to occur in a very short time, although we are not aware of any published studies. Nevertheless, stream communities may have difficulties tracking such rapid environmental cycles.

Logging, especially extensive clearcuts, increases soil loss at the basin, and increases siltation of the streambed[86,87,88,89]. This impact is detrimental to many organisms: algae are affected because of lack of stable substrate; mosses and macrophytes can die buried under the sediments; invertebrate communities change with their habitat; and gravel-spawning fishes like salmonids have lower reproductive success. We are studying some of these impacts of logging, but at this point we only have preliminary results. Nevertheless, they are consistent enough across regions[90,91], and suggest that frequent logging of eucalypt plantations can severely impact nearby streams.

Furthermore, logging can affect the morphology of stream channels, as well as their capacity to retain organic inputs. Trees from the riparian forests topple into stream channels, where fallen logs create debris dams, which are highly efficient retaining leaf litter, creating habitats for invertebrates as well as fish, and providing refuge during spates or droughts[92]. Research in the Agüera stream has shown that streams under frequently harvested forests (coppiced alder as well as eucalypt plantations) have a much lower abundance of logs and wood dams than reaches under mature deciduous forest[93,94]. Experimental removal of previously abundant woody debris in two reaches where this component was abundant[95] resulted in destabilization of the stream bed, scouring of fine sediments, dramatic shifts in habitat abundance, as well as changes in organic matter retention which could alter the energetic basis of the benthic community.

The studies by the Portuguese and Spanish research teams confirm the importance of forest practices in the structure and function of stream systems, and show some impacts associated with massive eucalypt plantations on the Iberian Peninsula. Also, they suggest that it is necessary to keep riparian buffer strips of native vegetation to reduce the human impact on streams and river. It is important to expand research in three directions: first, to increase our knowledge on the mechanisms mediating the response of different organisms to changes in litter inputs; second, to get a better picture of the local impacts of practices associated with forestry (tracks, wood-piles, clearing of understory, etc.); and third, to determine optimal management practices and soil uses at the landscape level.

## ACKNOWLEDGEMENTS

The research carried out in Portugal was financed by “Fundação para a Ciência” through several projects. The IMAR (Instituto do Mar–Centro Interdisciplinar de Coimbra) provided laboratory facilities. The Spanish group was founded by projects and grants by the University of the Basque Country, the Basque Government, and the Spanish Government. Comments from anonymous referees were greatly appreciated.

## BIOSKETCHES

**Manuel Graça** is a Lecturer in General and Animal Ecology, Department of Zoology, University of Coimbra, Portugal. He holds a Ph.D. in Zoology from the University of Sheffield, England. Dr. Graça's research interests fall into two areas: (a) decomposition of organic matter and (b) water quality of streams. He is interested in knowing the mechanisms ruling the decomposition of leaves in soil and freshwaters, particularly the relationship between leaf substrates, fungal decomposers and detritivores. He also has interest in understanding the ecological effects of deterioration of water quality in rivers and to optimise methods for the assessment of water quality using invertebrates as indicators.

**Cristina Canhoto** is Auxiliary lecturer in Ecology, Department of Zoology, University of Coimbra, Portugal. She holds a Ph.D. in Ecology from the University of Coimbra, Portugal. Dr. Canhoto has been investigating the trophic ecology of shredders in streams, particularly the role of plant chemical and physical defences on shredder invertebrates. She is also interested in understanding the chemical and physical changes of leaves during decomposition.

**Arturo Elosegi** is a Lecturer in Ecology and Limnology, Science Faculty, University of the Basque Country. He holds a Ph.D. in Biology from Universidad del País Basco, Spain. Dr. Elosegi's research focuses on function of Iberian streams, and modifications caused by human activities, especially forestry, livestock farming, and settlements. He has written papers on variation of water chemistry at different spatial and temporal scales, dynamics and retention of nutrients, stream metabolism, algal dynamics, leaf-litter budgets, and role of woody debris in streams.

**Jesus Pozo** is Professor in Ecology and Limnology, Faculty of Sciences, University of The Basque Country. He holds a Ph.D. in Biology from Universidad del País Basco, Spain. Dr. Pozo is interested in the study of the functioning of stream ecosystems, particularly the processing and fate of the terrestrial leaf-litter entering the streams, the organisms implicated in these processes, and the organic matter budgets. He also evaluates the repercussions of afforestation with exotic species on these natural processes.
